# Decomposition analysis of the decline in binary and triad undernutrition among preschool children in India

**DOI:** 10.1371/journal.pone.0292322

**Published:** 2023-10-20

**Authors:** Priyanka Dixit, Mayura Tonpe, Mrigesh Bhatia

**Affiliations:** 1 School of Health Systems Studies, Tata Institute of Social Sciences, Mumbai, India; 2 Dept. of Health Policy, London School of Economics, London, United Kingdom; Public Library of Science, UNITED STATES

## Abstract

**Background:**

To examine the socio-demographic factors associated with the decline in undernutrition among preschool children in India from National Family Health Survey (NFHS)-3, 2005–06 to NFHS– 5, 2019–21.

**Methods:**

For this study data were obtained from India’s nationally representative datasets such as NFHS-3 and NFHS-5. The outcome variables for this study were Binary undernutrition which were defined as the coexistence of anemia and either stunting or wasting and Undernutrition triad which were defined as the presence of Iron deficiency anemia, stunting and wasting, respectively. Decomposition analysis was used to study the factors responsible for a decline in undernutrition. This method was employed to understand how these factors contributed to the decline in undernutrition whether due to change in the composition (change in the composition of the population) or propensity (change in the health-related behaviour of the population) of the population over a period of 16 years.

**Results:**

Results showed that rate, which contributes 85.26% and 65.64%, respectively, to total change, was primarily responsible for a decline in both binary undernutrition and undernutrition triad. Reduction in Binary undernutrition was mainly explained by the change in the rate of education level of the mothers and media exposer during the inter-survey period. On the other hand, the decline in the Undernutrition triad can be explained by household wealth index, mother’s education, birth order and a change in people’s knowledge or practice about the preceding birth interval.

**Conclusion:**

Identifying important factors and understanding their relationship with the decline of undernutrition can be beneficial for reorienting nutrition-specific policies to achieve the targets of the Sustainable Development Goals by 2030.

## Background

Child undernutrition is a major public health problem that has adverse short and long-term health effects. It is not only an important risk factor for child mortality and morbidity globally, but it also often results in compromised cognitive development and physical capabilities, poor school performance, and low productivity [1].

Africa and Asia bear the greatest share of all forms of undernutrition. According to Global Nutrition Report, 2018 one-third of all malnourished children worldwide are from India, which has one of the highest rates of child malnutrition in the world [2]. The burden of under-nutrition among under-five children has not changed much even though various intervention programs are in operation in India. Nutrition indicators showed a slight reduction from NFHS-2015-16 to NFHS-2019-22, stunting has decreased from 38.4% to 35.5%, wasting from 21.0% to 19.3%, and the prevalence of underweight has decreased from 35.8% to 32.1%.

Wasting in children indicates acute undernutrition and is associated with a high risk of dying. Wasted children usually present with low weight-for-height or small mid-upper arm circumference. Stunting indicates chronic undernutrition, and stunted children have low height-for-age for measurements [3]. Although wasting responds rapidly to nutrition therapy, growth affected due to stunting is not amenable to rapid nutrition correction. As the etiology and processes associated with wasting and stunting are similar, the two forms are expected to often occur in the same child [4]. According to the Global Nutrition Report, about 16 million children worldwide are affected by both stunting and wasting [5]. Researchers have observed that the risk of mortality increases with combined anthropometric deficits such as stunting, wasting, and underweight [6].

Along with undernutrition, micronutrient deficiencies—such as those of iodine, iron, vitamin A, and zinc are of great public health concern, due to their high prevalence and the associated health and developmental consequences in children [7]. Iron deficiency is the most common form of micronutrient deficiency that persists with longer-lasting consequences such as impaired cognitive, behavioural, and motor development [7]. There is evidence to suggest that the cognitive deficits of iron deficiency may be irreversible, even if iron supplementation is begun within the critical period of zero to 24 months in children [8].

Globally, about 40 per cent of children under the age of five years suffer from iron deficiency anaemia. In India, there are about 59 per cent of children suffer from mild to severe degree anaemia [9, 10]. The co-occurrence of iron deficiency anaemia and undernutrition may have serious implications for children. Previous studies have examined the co-occurrence of undernutrition through the lens of the syndemic framework, focusing primarily on prevalence rates, socioeconomic characteristics, and income inequalities [11]. Existing literature has not sufficiently addressed the diverse socioeconomic and demographic factors that contribute to variations or change in the co-occurrence of undernutrition among children. In this study, the co-existence of “anemia & stunting” and, “anemia & wasting” was referred to as binary undernutrition, while the presence of “stunting, wasting, and anaemia” together was termed undernutrition triad.

To address the issue of Binary undernutrition and Undernutrition triad, policymakers must identify and understand the complex interplay of causative factors. Therefore, the main objective of this paper was to identify important factors in causing overall changes in the binary undernutrition and undernutrition triad. We also aimed to study how these factors were impacted by the change in composition (change in population structure) and propensity (change in knowledge or behaviour of population towards health care practices) of the population from the National Family Health Survey -3 (NFHS– 3), 2005–06 to NFHS– 5, 2019–21.

There are two crucial pathways that can change the overall Binary undernutrition and Undernutrition triad between NFHS– 3 and NFHS– 5. One could be a change in the shift in the proportion of children (compositional change) from one worse performing group (high prevalence of undernutrition) to a better performing group (low prevalence of undernutrition) since 2005–06. Another could be an increase in the probability of reduction of undernutrition (change in the propensity) among those subgroups which had higher rates of undernutrition during 2005–06 due to changes in the healthcare knowledge, behaviour, etc. To study these two pathways leading to a decline in undernutrition, decomposition analysis was performed. This analysis was performed using nationally-representative datasets thus contributing to the existing literature in understanding the enabiling causal mechanisms of Binary undernutrition and the Undernutrition triad in India.

## Methodology

For this study, individual‐level data were obtained from National Family Health Survey– 3 (NFHS– 3, 2005–06) and NFHS-5 (2019–2021) which were conducted under the stewardship of the Ministry of Health and Family Welfare (MOHFW), Government of India. International Institute for Population Sciences was designated as a nodal agency to conduct both surveys.

Both the surveys shared similar sampling design and data collection methods with a few exceptions. NFHS– 3 covered all 29 states and provided information about health indicators at the state and national level. On the other hand, NFHS –5 provided estimates of most indicators at the district level in the country as per the 2011 census of India. It included all six Union Territories along with 29 states for the first time [9, 12].

A two‐stage stratified multistage sampling design was used for both surveys. The census served as the sampling frame for the selection of the primary sampling units [9]. The Household questionnaire listed all usual members of the household and visitors who stayed in the household the night before the interview. Basic demographic information was collected on the characteristics of each person listed. The information on the age and sex of household members obtained in the Household questionnaire was used to identify women and men who were eligible for individual interviews.

Children’s information was extracted from selected households. From these households, eligible women (15–49 years) were selected and asked about the information of all the children specifically born during the last five years from the date of the survey. At the time of the survey height and weight of the child were recorded with the calibrated and standardized scale. Haemoglobin estimation of the children was done using the Dry Blood Sample method. Children aged 6–59 months with complete information including anthropometric measurement as well as haemoglobin estimation were included in the analysis of this study. The final sample size for NFHS– 3 and NFHS– 5 was 33,908 and 1,72,437 children at the India level respectively.

### I. Measures

#### A. Independent variables (Household, maternal, and child characteristics)

Many factors have significant effects on a child’s nutrition status. Based on the available literature, relevant variables were included in the model to adjust for the possible confounding effects of household, maternal, and child-level characteristics on the outcome variables. These variables are described as follows:

*a*. *Household characteristics*. We selected community-level factors such as–region of residence (North, Centre, East, North-East, West, and South) and place of residence (urban/rural).

Household-level variables like—religion [Hindu, Muslim, and Other religions (non-Hindu and non-Muslim)], caste [Scheduled Caste (SC) or Scheduled Tribe (ST), Other Backward Classes (OBC) and others (non-SC/ST and non-OBC)] and household size (<5, > = 5 members) were included in the study. Five categories of wealth Index—poorest, poorer, middle, richer, and richest were calculated based on household assets.

*b*. *Maternal characteristics*. Mother-level characteristics comprised variables like—the mother’s age in completed years (15–24, 25–34, and 35–49 years), level of education (No education, Primary, Secondary, and High school and above), Body mass Index (Thin, normal and overweight), and preceding birth interval (1st birth, < 24 months, and > 24 months).

The mother’s level of exposure to mass media (not exposed, partially exposed, and exposed) was assessed based on information about the frequency of reading a newspaper or magazine, listening to the radio, watching television, and visiting a cinema hall or theatre. Exposure to any one of the sources less than once a week and at least once a week was regarded as partially exposed. Contact with any mass media source was categorized as exposed and whereas no contact was regarded as not exposed.

*c*. *Children’s characteristics*. Child-level variables that were included in the study were—the age of the child in completed months (6–23, 24–35, and > 35 months), gender (Male and Female), birth order (1^st^, 2^nd^ - 4^th^, and 5^th^ or above), history of diarrhoea and fever in past two weeks (Yes and No).

#### B. Outcomes

The outcome variables in this study were the Binary undernutrition and Undernutrition triad, which were derived from the stunting, wasting, and anaemia status of children. As per WHO’s child growth standards, stunting was defined as height-for-age (HAZ), Z scores less than—2 Standard Deviation (SD) below the median height-for-age of the WHO reference population [13]. Wasting was defined as weight-for-height, Z-score less than −2 SD, and underweight as having a weight-for-age, Z-score less than −2 SD, compared with the respective median of the WHO reference population [13]. Anaemia was defined as a haemoglobin level of <11gm/dL in children.

The coexistence of undernutrition for this study was defined based on the co-occurrence of specified two, or three forms of undernutrition out of stunting, wasting and anaemia among children and were denoted as Binary undernutrition and Undernutrition triad respectively.

### II. Statistical methods

Bivariate analysis was carried out for cross-tabulating the outcome variables and selected background characteristics for both rounds of the survey (NFHS-3 and NFHS-5). Multivariable logistic regression technique was used to calculate the β coefficients and the adjusted odd’s ratios for the Binary undernutrition and Undernutrition triad for both datasets.

Data were analysed using the statistical analysis package STATA (Version 14.1 Stata Corporation, TX, USA). The analysis of the data was carried out after assigning survey weights that were available in the NFHS– 3 and NFHS– 5 datasets.

Multivariate decomposition analysis was carried out to study the role of different factors responsible for the decline in the Binary undernutrition and Undernutrition triad during NFHS– 3 and NFHS– 5. This study makes use of the mvdcmp module, which expands the traditional Oaxaca-Blinder decomposition to a number of nonlinear response models, including the logit model utilised here.

The mvdcmp method utilizes the results of regression models to divide decline in undernutretition into two components. The first component represents the decline that can be attributed to compositional change between groups, while the second component represents the decline resulting from differences in the effects of the characteristics or behavioral responses used as control variables [14, 15]. This method decompose the differences in the proportions of binary undernutrition and undernutrition triad, specifically focusing on the influence of compositional changes in households, as well as characteristics at the mother and child levels. The mvdcmp method is utilized to provide a detailed breakdown of the decomposition results. This approach not only reveals the composition and rate components within each variable but also examines their respective categories [15]. This procedure makes use of two components: composition and rate (propensity). Compositional change refers to structural changes in the population, such as a change in population literacy in two time periods or to a part of the overall change, which is ascribed to changes in the means of the covariates, keeping the rate as constant as it was in NFHS– 3. Rate or propensity changes stand for the change in the likelihood of undernutrition by different social, economic, and demographic sub-groups of the population as expressed by the β coefficients and constant terms of the binary regression, regardless of the change in the composition component. With the help of this method, we tried to determine the net contribution of each of the selected covariates to the declining trend of undernutrition (A detailed explanation of the decomposition formula can be found in S1 Appendix, Supplementary Method).

### III. Ethics

The data for this study was derived from NFHS– 3 and NFHS– 5 datasets which are available in the public domain and can be accessed through the Demographic and Health Surveys (DHS) Program website (https://dhsprogram.com/data/available-datasets.cfm). The available microdata is de-identified hence no identity of study participants can be revealed. For both surveys, written informed consent was obtained during the original data collection process before administering the questionnaire.

## Results

**Fig 1:**
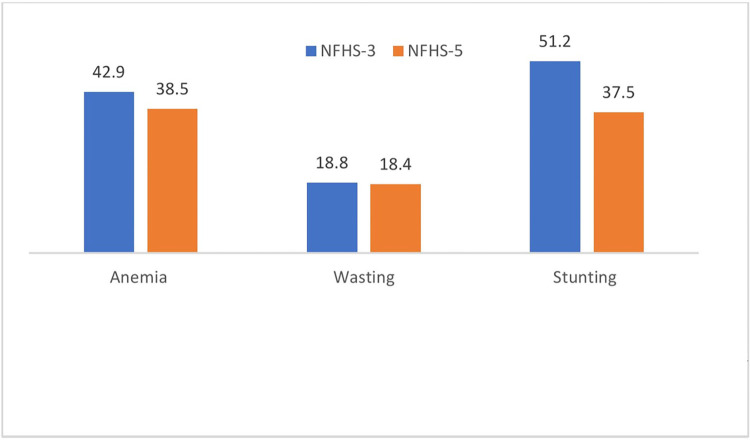
Prevalence of anemia, stunting and wasting among the children aged 6–59 months in India, NFHS-3 and 5.

Fig 1 shows prevalence of anemia, wasting and stunting from NFHS-3 to 5. It displays a decreasing trend in the prevalence of anemia, wasting, and stunting.

**Fig 2:**
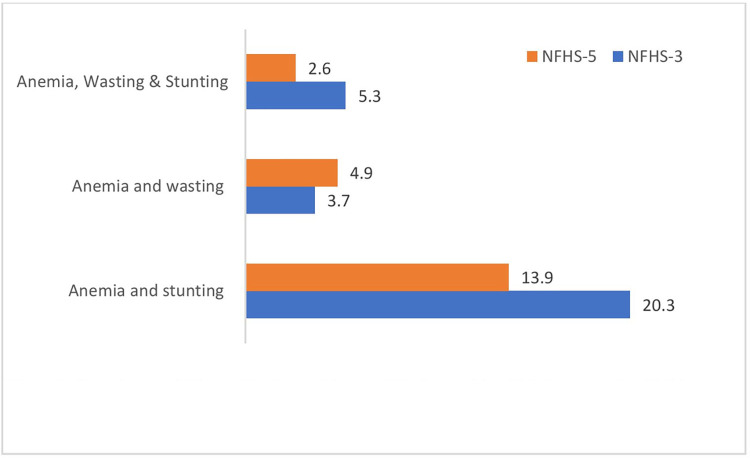
Prevalence of Binary undernutrition and Undernutrition tried among the children aged 6–59 months in India, NFHS-3 and 5. (S1 Table shows the profile of sample from NFHS-3 and NFHS-5).

Fig 2 shows the overall prevalence of coexistence of anemia and stunting, anemia and wasting and Undernutrition triad was found to be 20.3 per cent, 3.7 per cent and 5.3 respectively during NFHS– 3, whereas it was 13.9 per cent, 4.9 per cent and 2.6 respectively during NFHS– 5.

### I. Prevalence of Binary undernutrition and Undernutrition triad from NFHS– 3 to NFHS– 5

Table 1 displays the occurrence of Binary undernutrition (which includes the presence of anemia & stunting and anemia & wasting), as well as the Undernutrition triad, (which entails the presence of anemia, stunting, and wasting) in India across various socioeconomic and demographic factors in NFHS– 3 and NFHS– 5. The prevalence varied considerably by region, place of residence, wealth index, caste, religion, age of mother, mother’s educational status and degree of exposure to mass media, and child’s characteristics like age, birth order, gender, birth interval etc. Stunting and anaemia were more prevalent in the central region during both NFHS rounds, while anaemia wasting and undernutrition tried were more prevalent in the eastern region during the first round and the western region during the second. Both Binary undernutrition and the Undernutrition triad were higher among those living in rural areas, from the poorest households, those belonging to SC/ST caste, and also among children from thin BMI mothers. During the third and fifth rounds of NFHS, children with mothers lacking education, limited exposure to mass media, and birth intervals of less than two years exhibited higher prevalence of both Binary undernutrition and the Undernutrition triad.

**Table 1:** Prevalence of Binary undernutrition and Undernutrition triad among 6–59 –month-old children according to selected background characteristics in India, NFHS– 3 (2005–06) & NFHS– 5 (2019–21).

	NFHS-3			NFHS-5		
Background Variables	Anaemia and stunting	Anaemia and wasting	Anaemia, Wasting, Stunting	Anaemia and stunting	Anaemia and wasting	Anaemia, Wasting, Stunting
**Region**						
North	19.1	3.6	4.8	12.9	4.2	1.5
Central	25.7	3.7	5.8	15.9	4.3	2.4
East	18.3	4.2	7.2	13.9	5.4	3.2
Northeast	16.2	2.9	3.2	11.0	4.4	1.6
West	21.2	2.7	4.1	14.9	7.1	4.4
South	15.4	3.7	2.9	11.4	3.8	2.0
**Place of Residence**						
Urban	16.3	3.1	3.0	11.3	4.5	2.0
Rural	21.6	3.8	6.0	14.8	5.0	2.9
**Wealth Index**						
Poorest	25.8	4.4	7.9	18.5	5.8	4.3
Poorer	22.7	3.7	7.2	15.9	5.1	2.8
Middle	21.0	3.7	4.6	13.1	4.6	2.4
Richer	17.4	3.2	3.3	11.0	4.4	1.8
Richest	9.4	2.6	1.2	8.3	3.9	1.2
**Religion**						
Hindu	20.6	3.6	5.5	14.1	4.9	2.8
Muslim	19.8	3.8	4.7	13.7	5.1	2.2
Others	16.9	3.5	4.6	10.6	3.9	1.9
**Caste**						
SC/ST	24.1	4.4	7.2	16.6	5.6	3.4
OBC	20.8	3.4	5.4	13.3	4.5	2.5
Others	15.7	3.2	3.2	11.1	4.5	1.9
**Household member size**						
<5	17.6	3.3	4.5	12.5	4.9	2.7
5+	21.1	3.8	5.5	14.4	4.8	2.6
**Mothers education level**						
No Education	24.9	4.0	7.4	19.2	5.5	3.8
Primary	19.3	3.8	5.0	16.7	5.0	3.4
Secondary	15.7	3.2	2.7	12.8	4.9	2.3
Higher	6.8	2.7	1.4	7.9	3.8	1.4
**Mother current age (in years)**						
15–24	21.2	4.1	5.7	15.6	5.4	3.0
25–34	19.6	3.4	4.9	13.2	4.7	2.5
35–49	20.3	3.2	6.0	13.1	4.3	2.4
**Body Mass Index**						
Thin	21.2	4.7	7.4	17.4	6.3	4.9
Normal	20.6	3.2	4.3	14.0	5.0	2.4
Overweight	12.7	1.4	0.9	10.0	2.9	1.2
**Media Exposure**						
No Exposure	24.1	3.9	7.1	16.3	5.3	3.2
Partial	17.8	3.6	3.9	11.6	4.5	2.1
Complete	9.8	1.9	2.2	8.5	3.9	1.5
**Preceding Birth Interval (months)**						
First birth	16.8	3.4	3.8	11.6	4.5	2.2
<24	24.8	3.3	5.7	17.8	5.0	3.4
= >24	20.7	3.9	6.0	14.4	5.1	2.7
**Age of the child (in months)**						
6–23	21.5	6.9	8.1	16.9	7.7	3.4
24–35	25.2	2.7	5.9	16.3	5.2	2.8
>36	17.1	1.9	3.1	10.8	2.9	2.1
**Sex of Child**						
Male	19.8	3.6	6.0	13.9	4.9	2.9
Female	20.9	3.8	4.5	13.8	4.8	2.3
**Birth Order**						
First Order	16.8	3.4	3.8	11.6	4.5	2.2
2–4	20.5	3.8	5.4	14.9	5.1	2.9
More than 5	26.0	3.5	7.8	20.1	5.2	3.7
**Diarrhoea**						
No	20.1	3.5	5.1	13.7	4.8	2.6
Yes	22.4	5.6	7.4	16.0	6.1	3.8
**Fever**						
No	20.3	3.5	5.0	13.8	4.7	2.6
Yes	20.4	4.5	7.0	14.6	5.9	3.1

### II. Multivariable binary logistic regression for Binary undernutrition and Undernutrition tried during NFHS– 3 and NFHS– 5

To enhance the understanding of the role of different household, maternal, and child characteristics on the Binary undernutrition & undernutrition triad in India the result of the multivariable binary logistic regression model has been described in Table 2. This table showed that the simultaneous presence of anemia and stunting was high among the wastern region compare to north region. Conversely, the odds of this dual condition was lower among residents of the east and north-east regions when compared to those living in the north region. This pattern held true for both survey periods. It was observed that there was a greater decline in Binary undernutrition between two surveys among children from the richest group. Education and the increasing age of mothers demonstrated an inverse relationship with prevalence of anemia and stunting. Compared to thin BMI mothers, normal and overweight BMI mother’s children were less likely to experience coexistence of anemia and wasting and undernutried tried in both the survey. The occurrence of Binary undernutrition among children who had suffered from diarrhoea and fever in the recent past became less pronounced during NFHS– 5 as compared to NFHS– 3.

**Table 2:** Multivariable binary logistic regression for Binary undernutrition and Undernutrition triad among 6–59 month-old children in India, NFHS– 3 & NFHS– 5.

	NFHS-3			NFHS-5		
Background Variables	Anaemia and stunting	Anaemia and wasting	Anaemia, Wasting & Stunting	Anaemia and stunting	Anaemia and wasting	Anaemia, Wasting & Stunting
**Region (North)** ^**R**^						
Central	1.172***(1.071 1.282)	1.026 (0.845 1.245)	1.016 (0.856 1.205)	1.018 (0.974 1.065)	0.86***(0.799 0.925)	1.2***(1.069 1.345)
East	0.717***(0.648 0.794)	1.02 (0.831 1.251)	1.075 (0.899 1.284)	0.779***(0.742 0.818)	0.973 (0.902 1.05)	1.314***(1.169 1.477)
Northeast	0.596***(0.528 0.673)	0.707***(0.548 0.914)	0.567***(0.443 0.725)	0.628***(0.59 0.668)	0.631***(0.571 0.697)	0.678***(0.576 0.798)
West	1.155***(1.032 1.293)	0.735***(0.566 0.956)	0.981 (0.781 1.232)	1.166***(1.103 1.233)	1.636***(1.51 1.773)	2.653***(2.348 2.996)
South	0.814***(0.728 0.91)	1.236**(0.994 1.538)	0.795***(0.634 0.997)	0.978 (0.925 1.034)	0.885***(0.809 0.968)	1.499***(1.307 1.718)
**Place of Residence (Urban)** ^**R**^						
Rural	0.865***(0.802 0.933)	1.055 (0.899 1.237)	0.972 (0.836 1.129)	0.965 (0.926 1.007)	0.984 (0.923 1.051)	0.913**(0.827 1.008)
**Wealth Index (Poorest)** ^**R**^						
Poorer	0.946 (0.866 1.033)	0.783***(0.646 0.949)	1.011 (0.872 1.172)	0.903***(0.868 0.938)	0.888***(0.833 0.946)	0.77***(0.708 0.839)
Middle	0.821***(0.745 0.904)	0.837**(0.682 1.029)	0.732***(0.613 0.875)	0.788***(0.752 0.826)	0.849***(0.788 0.915)	0.702***(0.632 0.779)
Richer	0.681***(0.608 0.761)	0.844 (0.667 1.068)	0.653***(0.528 0.808)	0.71***(0.672 0.75)	0.831***(0.762 0.906)	0.544***(0.477 0.62)
Richest	0.426***(0.368 0.494)	0.865 (0.644 1.161)	0.343***(0.248 0.473)	0.585***(0.545 0.627)	0.726***(0.651 0.809)	0.467***(0.392 0.556)
**Religion (Hindu)** ^**R**^						
Muslim	1.011 (0.928 1.102)	1.059 (0.884 1.269)	0.92 (0.777 1.091)	1.053***(1.007 1.1)	1.267***(1.185 1.354)	1.051 (0.948 1.166)
Others	0.859***(0.761 0.969)	0.892 (0.696 1.143)	0.96 (0.761 1.211)	0.791***(0.745 0.84)	0.724***(0.656 0.799)	0.751***(0.648 0.872)
**Caste (SC/ST)** ^**R**^						
OBC	0.929**(0.864 1)	0.837***(0.717 0.977)	0.833***(0.73 0.95)	0.867***(0.839 0.897)	0.864***(0.819 0.912)	0.856***(0.795 0.921)
Others	0.781***(0.718 0.85)	0.807***(0.678 0.961)	0.677***(0.575 0.798)	0.79***(0.756 0.826)	0.892***(0.833 0.954)	0.752***(0.677 0.835)
**Household member size (<5)** ^**R**^						
5+	1.106***(1.026 1.193)	1.044 (0.897 1.216)	0.983 (0.853 1.132)	1.057***(1.022 1.093)	0.924***(0.877 0.973)	0.942 (0.875 1.015)
**Mothers education level (Illiterate)** ^**R**^						
Primary	0.884***(0.809 0.965)	0.908 (0.751 1.097)	0.879 (0.748 1.033)	0.923***(0.882 0.966)	0.89***(0.824 0.962)	0.911**(0.826 1.005)
Secondary	0.785***(0.722 0.853)	0.842**(0.706 1.005)	0.611***(0.516 0.723)	0.761***(0.732 0.79)	0.914***(0.859 0.972)	0.74***(0.681 0.804)
Higher	0.474***(0.385 0.582)	0.73**(0.509 1.047)	0.593***(0.382 0.92)	0.6***(0.563 0.639)	0.786***(0.714 0.866)	0.566***(0.484 0.661)
**Mother current age (in years) (15–24)** ^**R**^						
25–34	0.853***(0.794 0.917)	1.002 (0.864 1.161)	0.96 (0.838 1.1)	0.863***(0.834 0.894)	1.082***(1.024 1.144)	0.951 (0.88 1.028)
35–49	0.693***(0.608 0.791)	1.08 (0.814 1.432)	0.983 (0.775 1.246)	0.778***(0.732 0.827)	1.159***(1.052 1.277)	0.903 (0.787 1.037)
Body Mass Index (Thin) ^**R**^						
Normal	1.001 (0.941 1.066)	0.7***(0.616 0.795)	0.613***(0.548 0.686)	0.855***(0.826 0.885)	0.883***(0.836 0.932)	0.613***(0.572 0.657)
Overweight	0.951 (0.834 1.085)	0.42***(0.305 0.579)	0.286***(0.194 0.424)	0.749***(0.713 0.787)	0.625***(0.576 0.678)	0.385***(0.339 0.437)
**Media Exposure (**No Exposure**)** ^**R**^						
Partial	0.911***(0.849 0.978)	0.945 (0.812 1.098)	0.904 (0.794 1.029)	0.942***(0.911 0.973)	0.978 (0.928 1.03)	0.914***(0.847 0.986)
Complete	0.688***(0.584 0.811)	0.672***(0.48 0.941)	1.077 (0.777 1.493)	0.915 (0.788 1.063)	0.998 (0.803 1.241)	0.965 (0.675 1.382)
**Birth Interval (first birth)** ^**R**^						
<24	1.337 (0.627 2.848)	0.829 (0.198 3.466)	0.284***(0.108 0.744)	0.974 (0.739 1.284)	0.893 (0.577 1.383)	0.595**(0.351 1.008)
>24	1.063 (0.5 2.262)	0.951 (0.228 3.96)	0.275***(0.105 0.718)	0.814 (0.618 1.072)	0.926 (0.599 1.429)	0.508***(0.301 0.859)
**Age of the child (6–23 months)** ^**R**^						
24–35	1.148***(1.063 1.239)	0.43***(0.365 0.508)	0.781***(0.68 0.896)	0.925***(0.891 0.959)	0.682***(0.645 0.721)	0.794***(0.732 0.862)
>36	0.701***(0.653 0.753)	0.287***(0.247 0.334)	0.435***(0.381 0.497)	0.607***(0.587 0.628)	0.367***(0.347 0.387)	0.573***(0.532 0.618)
**Sex of Child (Male)** ^**R**^						
Female	1.009 (0.951 1.069)	1.036 (0.917 1.17)	0.739***(0.662 0.826)	0.959***(0.933 0.986)	0.961**(0.92 1.005)	0.742***(0.697 0.791)
**Birth Order (First birth)** ^**R**^						
2–4	1.048 (0.492 2.23)	1.203 (0.289 5.009)	4.41***(1.685 11.542)	1.424***(1.081 1.876)	1.131 (0.733 1.747)	2.278***(1.347 3.852)
More than 5	1.252 (0.585 2.68)	0.901 (0.213 3.817)	4.923***(1.853 13.074)	1.702***(1.283 2.257)	1.004 (0.642 1.571)	2.634***(1.533 4.526)
**Diarrhoea (No)** ^**R**^						
Yes	1.101**(0.997 1.216)	1.236***(1.03 1.482)	1.154**(0.974 1.367)	1.042 (0.987 1.1)	1.034 (0.952 1.122)	1.154***(1.033 1.289)
**Fever (No)** ^**R**^						
Yes	0.953 (0.877 1.035)	1.135 (0.968 1.331)	1.273***(1.106 1.465)	1.054***(1.01 1.099)	1.144***(1.074 1.22)	1.119***(1.022 1.225)

Note: R: Indicate reference catagory

The results indicated that the prevalence of coexisting anemia and wasting, as a form of undernutrition, was significantly lower among children from the north-east area compared to children from the north region during both survey periods. In NFHS-3, children from the western region had a lower likelihood of experiencing both anemia and wasting compared to children from the north. However, in NFHS-5, there was a shift, and children from the western region were more likely to experience the coexistence of anemia and wasting compared to children from the north. In both time periods, children from poorer and middle-class households exhibited a lower likelihood of experiencing both anemia and wasting compared to children from the poorest households. Non-SC/ST children demonstrated a lower likelihood of being undernourished during both time periods, as compared to SC/ST children. Moreover, children of mothers with a secondary education or higher, a normal BMI, and who were overweight had a considerably lower risk of being wasted and anaemic in both the survey period. In NFHS-3, children who had recently experienced diarrhea, and in NFHS-5, children who had recently had a fever, showed a higher likelihood of having binary undernutrition i.e. anemia, and wasting compared to children who did not exhibit these medical symptoms.

In Table 2 the results of the multivariable binary logistic regression model are depicted to understand the role of different covariates of the Undernutrition triad i.e. coexistence of anemia, stunting and wasting together at two-survey points of time. Unlike previous survey, in NFHS-5, the undernutrition triad was more likely to occur among children from all geographical regions except for those from the North-East. In NFHS-5, children from urban areas had higher odds of undernutrition triad than those from rural areas. An inverse relationship was noticed between the undernutrition triad and the wealth index of children. As the wealth index of the children increased, they were less likely to face the undernutrition triad.

Households that belonged to OBC and other castes, had significantly less chance to have children with the undernutrition triad. Children born to thin and overweight mothers were more likely to be undernourished. In NFHS-5, children whose mothers had partial exposure to media played a significant role and had a decreased likelihood of experiencing all three stages of undernutrition. Gender differences were seen to be significantly associated with the undernutrition triad. The undernutrition triad was found to be less likely among girls than boys. Diarrhoea and fever were found to be significantly associated with the undernutrition triad despite the 16-year gap between the two surveys.

According to the descriptive and regression results, compositional changes as well as changes in socioeconomic and demographic factors may have contributed to the significant drop in Binary i.e. anemia and stunting and triad undernutrition in India from NFHS-3 to 5. We could detect a little escalation in the simultaneous presence of wasting and anaemia. Therefore, decomposition analysis were based on only two outcome variables, 1) binary undernutrition as indicated by “anemia & stunting” and 2) Undernutrition tried, as indicated by the presence of “anemia, stunting, and wasting”.

### III. Decomposition of change in the Binary undernutrition by using multivariable binary regression model in India, 2005–06 and 2019–21

Table 3 describes the decomposition of the overall decline in the binary undernutrition into different components, namely—composition and the rate at aggregate and sub-group levels by using multivariable binary logistic regression. Table 3 displays the findings on the contributions of changes in each of the variable categories as well as overall in terms of both the composition and rate components. The composition effect (absolute and percentage), rate effect (absolute and percentage), and total effect (the sum of the absolute and percentage effects of both composition and rate components) data are shown in Table 3.

**Table 3:** Decomposition of change in Binary undernutrition by using multivariable binary logistic regression model in India, NFHS– 3 & NFHS– 5.

Binary undernutrition (Stunting and anaemia)	Compositional (Endowment) Effect			Rate Effect			Total Effect	
	Absolute	P value	Percentage	Absolute	P value	Percentage	Absolute	Percentage
**Total**	0.00495	0.042	14.74	0.02862	0.000	85.26	0.03357	100.00
**Household level factors**								
**Region**								
North	0.00047	0.701	1.41	0.00082	0.405	2.45	0.00129	3.86
Central	-0.0037	0.697	-11.04	0.00609	0.000	18.15	0.00239	7.11
East	0.00353	0.696	10.51	-0.00147	0.176	-4.38	0.00206	6.13
Northeast	0.00064	0.697	1.90	-0.00047	0.679	-1.41	0.00017	0.49
West	0.00381	0.697	11.36	0.00028	0.628	0.85	0.00409	12.21
South	-0.0011	0.707	-3.29	-0.00271	0.001	-8.08	-0.00381	-11.37
Total			10.85			7.58		18.43
**Place of Residence**								
Urban	0.00914	0.693	27.24	0.00158	0.014	4.70	0.01072	31.94
Rural	0.00914	0.693	27.24	-0.00629	0.014	-18.73	0.00285	8.51
Total			54.48			-14.03		40.45
**Wealth Index**								
Poorest	-0.02069	0.701	-61.63	0.00207	0.190	6.17	-0.01862	-55.46
Poorer	-0.01027	0.701	-30.6	0.00338	0.005	10.08	-0.00689	-20.52
Middle	0.00080	0.702	2.37	0.00264	0.005	7.87	0.00344	10.24
Richer	-0.00367	0.703	-10.94	0.00030	0.738	0.88	-0.00337	-10.06
Richest	-0.03413	0.700	-101.67	-0.00485	0.000	-14.46	-0.03898	-116.13
Total			-202.47			10.54		-191.93
**Religion**								
Hindu	-0.00044	0.699	-1.31	-0.00149	0.627	-4.44	-0.00193	-5.75
Muslim	0.00101	0.711	3.02	-0.00108	0.158	-3.22	-0.00007	-0.20
Others	0.00085	0.702	2.52	0.00125	0.146	3.72	0.00210	6.24
Total			4.23			-3.94		0.29
**Caste**								
SC/ST	-0.00735	0.702	-21.9	-0.00113	0.453	-3.37	-0.00848	-25.27
OBC	-0.00115	0.714	-3.43	0.00270	0.038	8.05	0.00155	4.62
Others	-0.01448	0.703	-43.15	-0.00092	0.281	-2.76	-0.0154	-45.91
Total			-68.48			1.92		-66.56
**Household member size**								
<5	0.00102	0.700	3.03	-0.00084	0.286	-2.52	0.00018	0.51
5+	0.00102	0.700	3.03	0.00238	0.286	7.09	0.00340	10.12
Total			6.06			4.57		10.63
**Mother Level factors**								
**Mothers education level**								
No education	0.04029	0.687	120.04	0.00192	0.129	5.72	0.04221	125.76
Primary	0.00173	0.687	5.15	0.00031	0.701	0.93	0.00204	6.08
Secondary	-0.00402	0.717	-11.97	0.00693	0.010	20.66	0.00291	8.69
Higher	0.02136	0.688	63.64	-0.00325	0.039	-9.69	0.01811	53.95
Total			176.86			17.62		194.48
**Mother current age (in years)**								
15–24	0.01007	0.693	30.00	0.00170	0.216	5.06	0.01177	35.06
25–34	-0.00083	0.740	-2.49	0.00266	0.249	7.92	0.00183	5.43
35–49	0.00116	0.694	3.46	-0.00108	0.107	-3.21	0.00008	0.25
Total			30.97			9.77		40.74
**Body Mass Index**								
Thin	0.00189	0.683	5.63	-0.00358	0.000	-10.67	-0.00169	-5.04
Normal	-0.00089	0.716	-2.67	0.00226	0.359	6.74	0.00137	4.07
Overweight	0.00219	0.680	6.53	0.00278	0.016	8.28	0.00497	14.81
Total			9.49			4.35		13.84
**Media Exposure**								
No exposure	-0.01819	0.707	-54.20	0.00826	0.011	24.6	-0.00993	-29.6
Partial	0.00396	0.712	11.79	0.00518	0.050	15.43	0.00914	27.22
Complete	-0.01198	0.707	-35.71	-0.00033	0.010	-0.99	-0.01231	-36.7
Total			-78.12			39.04		-39.08
**Child level Factors**								
**Preceding Birth Interval (months)**								
First birth	0.00562	0.766	16.75	-0.01055	0.478	-31.43	-0.00493	-14.68
<24	0.00282	0.708	8.40	0.00281	0.381	8.36	0.00563	16.76
>24	-0.00179	0.773	-5.34	0.00473	0.600	14.1	0.00294	8.76
Total			19.81			-8.97		10.84
**Age of the child (in months)**								
6–23	0.00084	0.699	2.51	-0.0051	0.000	-15.18	-0.00426	-12.67
24–35	-0.00004	0.697	-0.11	0.00312	0.000	9.3	0.00308	9.19
>36	0.00325	0.697	9.69	0.00163	0.296	4.87	0.00488	14.56
Total			12.09			-1.01		11.08
**Sex of Child**								
Male	-0.00003	0.818	-0.10	-0.00185	0.128	-5.51	-0.00188	-5.61
Female	-0.00003	0.818	-0.10	0.00174	0.128	5.19	0.00171	5.09
Total			-0.20			-0.32		-0.52
**Birth Order**								
First order	0.00434	0.792	12.94	0.01104	0.456	32.89	0.01538	45.83
2–4	0.0004	0.797	1.19	-0.00819	0.460	-24.39	-0.00779	-23.2
More than 5	0.00766	0.711	22.81	-0.00087	0.471	-2.61	0.00679	20.2
Total			36.94			5.89		42.83
**Diarrhoea**								
No	0.00087	0.700	2.59	-0.00366	0.342	-10.9	-0.00279	-8.31
Yes	0.00087	0.700	2.59	0.00027	0.342	0.8	0.00114	3.39
Total								
**Fever**								
No	-0.0005	0.716	-1.48	0.00626	0.035	18.65	0.00576	17.17
Yes	-0.0005	0.716	-1.48	-0.00092	0.035	-2.74	-0.00142	-4.22
Total			2.22			5.81		8.03
**Intercept**								6.47

This table highlights that the major component responsible for the decline in binary undernutrition was rate, which accounted for about 85.26 per cent and contribution of composition was 14.74 percent of the overall change. The decomposition analysis revealed that the composition effects of all factors were found to be statistically insignificant. Among the total rate contribution of 100 percent, household variables accounted for 6.64 percent, mother-level factors contributed to 70.78 percent, and child-level factors contributed to 1.4 percent. The contributions of each variable category highlight that the reduction in binary undernutrition in India can be primarily attributed to changes in the rates of rural residence (-18.73%), the richest (-14.46%), middle (-14.46%) wealth index category (7.87%), mothers with secondary level education (20.66%), partial mass media exposure (-15.18%), first birth (-31.43%), child age (-15.18%), birth interval less than 24 months (-24.39%), and the occurrence of fever (18.65%). In terms of mother education and mass media exposer, the rate effect is 17.62 and 39.04 percent which corresponded to 194.48 and -39.08 percent respectively to the total change in binary undernutrition.

### IV. Decomposition of change in the Undernutrition triad by using multivariable binary regression model in India, 2005–06 and 2019–21

Table 4 shows the decomposition of the overall decline in the undernutrition triad into different components namely—rate and composition, at aggregate and sub-group levels by using multivariable binary logistic regression. The results of the overall multivariate decomposition analysis showed that the differences in endowment or composition across surveys accounted for about 34.36% of the overall decrease in undernutrition over the past 16 years, while the differences in coefficient across surveys accounted for the remaining 65.64%. The table highlights that various factors, such as the central and northeast regions, households classified as the poorest and richest, SC/ST and other castes, mothers with secondary education or lower, thin and overweight BMIs, the age of the child, birth order, and recent episodes of fever, all emerged as significant contributors in the composition. With the exception of the north-eastern, all five regions and various factors such as household wealth (both poor and richest), religious affiliation (Muslim and other religions), and the age of children played a significant role in the observed decline of undernutrition. Among household variables, the contribution of endowment was -45.78, while the rate was 24.64, resulting in a combined contribution of -21.14 percent decline during the inter-survey period. Regarding mother-level factors, 66.98 percent of the decline can be attributed to composition, while -8.48 percent is due to the rate, resulting in a combined contribution of 58.5 percent. In contrast to household and mother-level factors, the contribution of child-level factors to the overall decline in nutrition was relatively less. Composition accounted for 13.13 percent, while the rate contributed -10.80 percent, resulting in a combined contribution of only 2.33 percent.

**Table 4:** Decomposition of change in Undernutrition tried by using multivariable binary logistic regression model in India, NFHS– 3 & NFHS– 5.

Stunting, Wasting and anaemia	Compositional Effect (Explained part)			Rate Effect (Unexplained part)			Total Effect	
	Absolute	P value	Percentage	Absolute	P value	Percentage	Absolute	Percentage
Total change	0.00611	0.000	34.36	0.01168	0.000	65.64	0.01779	100.00
**Household level factors**								
**Region**	0.00003	0.091	0.17	0.00055	0.002	13.17	0.00058	13.34
North	-0.00012	0.03	-0.67	0.0017	0.004	9.57	0.00158	8.90
Central	-0.00022	0.005	-1.23	0.00112	0.023	6.27	0.00090	5.04
East	0.00005	0.001	0.27	0.0010	0.094	5.64	0.00105	5.91
Northeast	0.0001	0.231	0.54	-0.00199	0.000	-11.19	-0.00189	-10.65
West	-0.0001	0.216	-0.55	-0.00119	0.006	-6.67	-0.00129	-7.22
Total			-1.47			16.79		15.32
**Place of Residence**								
Urban	0.00012	0.695	0.700	-0.00022	0.504	-1.21	-0.0001	-0.51
Rural	0.00012	0.695	0.700	0.00086	0.504	4.81	0.00098	5.51
Total			1.400			3.60		5.00
**Wealth Index**								
Poorest	-0.00166	0.001	-9.31	-0.00039	0.598	-2.19	-0.00205	-11.5
Poorer	-0.00104	0.000	-5.86	0.00191	0.002	10.76	0.00087	4.90
Middle	0.00002	0.462	0.14	0.00003	0.955	0.15	0.00005	0.29
Richer	-0.00018	0.341	-1.03	0.00087	0.072	4.87	0.00069	3.84
Richest	-0.00291	0.001	-16.35	-0.00156	0.014	-8.78	-0.00447	-25.13
Total			-32.41			4.81		-27.6
**Religion**								
Hindu	-0.00003	0.412	-0.15	-0.00095	0.537	-5.35	-0.00098	-5.5
Muslim	-0.00005	0.533	-0.28	-0.00084	0.036	-4.7	-0.00089	-4.98
Others	0.0000	0.995	0.000	0.00093	0.036	5.23	0.00093	5.23
Total			-0.43			-4.82		-5.25
**Caste**								
SC/ST	-0.00088	0.001	-4.93	0.00063	0.384	3.54	-0.00025	-1.39
OBC	-0.00002	0.85	-0.10	0.00022	0.731	1.23	0.0002	1.13
Others	-0.00137	0.004	-7.70	-0.00044	0.32	-2.50	-0.00181	-10.2
Total			-12.73			2.27		-10.46
**Household member size**								
<5	-0.00001	0.81	-0.07	-0.00019	0.604	-1.09	-0.0002	-1.16
5+	-0.00001	0.81	-0.07	0.00055	0.604	3.08	0.00054	3.01
Total			-0.14			1.99		1.85
**Mother Level factors**								
**Mothers education level**								
No education	0.00272	0.000	15.27	0.0003	0.629	1.70	0.00302	16.97
Primary	0.00012	0.033	0.65	0.00002	0.956	0.12	0.00014	0.77
Secondary	0.00153	0.015	8.57	-0.00266	0.056	-14.93	-0.00113	-6.36
Higher	0.00069	0.120	3.90	0.00048	0.545	2.67	0.00117	6.57
Total			28.39			-10.44		17.95
**Mother current age (in years)**								
15–24	0.00007	0.732	0.41	-0.00032	0.625	-1.77	-0.00025	-1.36
25–34	0.00007	0.623	0.41	-0.00047	0.665	-2.62	-0.0004	-2.21
35–49	0.0000	0.975	-0.01	0.0002	0.523	1.1	0.0002	1.09
Total			0.81			-3.29		-2.48
**Body Mass Index**								
Thin	0.00451	0	25.35	0.00065	0.243	3.67	0.00516	29.02
Normal	-0.00031	0.161	-1.74	0.00215	0.229	12.11	0.00184	10.37
Overweight	0.00291	0.000	16.37	-0.00125	0.194	-7.03	0.00166	9.34
Total			39.98			8.75		48.73
**Media Exposure**								
No exposure	-0.00024	0.646	-1.37	-0.00017	0.92	-0.96	-0.00041	-2.33
Partial	-0.0003	0.184	-1.71	-0.00047	0.746	-2.63	-0.00077	-4.34
Complete	0.00016	0.679	0.88	0.00002	0.818	0.09	0.00018	0.97
Total			-2.20000			-3.50000		-5.7
**Child level Factors**								
**Preceding Birth Interval (months)**								
First birth	-0.00275	0.019	-15.43	0.00605	0.232	34.02	0.0033	18.59
<24	-0.00045	0.026	-2.51	-0.00164	0.141	-9.21	-0.00209	-11.72
>24	-0.00094	0.017	-5.3	-0.00264	0.392	-14.81	-0.00358	-20.11
Total			-23.24			10.00		-13.24
**Age of the child (in months)**								
6–23	0.00028	0.000	1.58	0.00102	0.041	5.73	0.0013	7.31
24–35	0.0000	0.018	-0.01	0.00064	0.115	3.60	0.00064	3.59
>36	0.00036	0.000	2.04	-0.00299	0.001	-16.79	-0.00263	-14.75
Total			3.61			-7.46		-3.85
**Sex of Child**								
Male	0.00008	0.000	0.45	0.00004	0.95	0.21	0.00012	0.66
Female	0.00008	0.000	0.45	-0.00003	0.95	-0.19	0.00005	0.26
Total			0.90			0.02		0.92
**Birth Order**								
First order	0.00328	0.006	18.44	-0.00569	0.261	-31.99	-0.00241	-13.55
2–4	-0.00028	0.014	-1.58	0.00458	0.23	25.77	0.0043	24.19
5^th^ and Higher	0.00216	0.004	12.16	0.00041	0.332	2.32	0.00257	14.48
Total			29.02			-3.9		25.12
**Diarrhoea**								
No	0.00009	0.113	0.49	-0.00001	0.997	-0.03	0.00008	0.46
Yes	0.00009	0.113	0.49	0.0000	0.997	0.00	0.00009	0.49
Total			0.98			-0.03		0.95
**Fever**								
No	0.00017	0.004	0.93	-0.00197	0.14	-11.05	-0.0018	-10.12
Yes	0.00017	0.004	0.93	0.00029	0.14	1.62	0.00046	2.55
Total			1.86			-9.43		-7.57
Intercept								60.27

The primary factor driving the reduction in the undernutrition triad was identified as the change in mothers’ BMI, accounting for approximately 49 percent. Household wealth index contributed -28 percent, and child birth order accounted for 25 percent. Notably, for all these variables, the composition effect outweighed the rate effect.

## Discussion

Child undernutrition—a serious public health problem, is a leading cause of concern globally. In the present study, we observed the trend of the Binary undernutrition and Undernutrition triad among children and important correlates associated with the change using nationally representative datasets such as NFHS– 3 and NFHS– 5. Our results showed that the prevalence of Binary undernutrition based on anemia and stunting and Undernutrition triad declined over the last 16 years between the two surveys. The decline in undernutrition may result from a decrease in the occurrence of undernutrition among children from the poorest and middle wealth quintiles or a change in the population composition of the same group from poor to rich wealth quintiles. The central finding of this paper is that the major components responsible for the decline in the Binary undernutrition and Undernutrition triad were rate effect, which accounted for about 85.26 per cent and 65.64 per cent of the overall change respectively. In binary undernutrition, the influence of factors under composition was not found to be significant. However, when considering undernutrition cases holistically and examining multiple factors together, it was observed that factors related to composition contribute approximately 34.36 percent to the overall undernutrition decline.

Previous studies have documented regional variations in all forms of undernutrition, which may be due to contextual factors such as social environment, shared cultural and social norms, feeding practices, place of delivery, and variations in local implementation of nutrition-specific policies [16–19]. Our findings showed that compared to the Northern region, the Central region in NFHS-3, and Western region in NFHS-3 and 5 regions had higher Binary undernutrition. Higher trends of undernutrition in the Central and Western regions of the country have been documented in past studies [1, 20]. Researchers suggested that factors such as poverty, illiteracy, lack of economic development and political commitment, maternal undernutrition, etc. may be involved in a higher prevalence of undernutrition in these regions [1]. This persisting regional trend highlights the need for more intensive and need-based nutrition interventions in the Central and Western regions of India.

One of the major contributing factors to the reduction in the Binary undernutrition and Undernutrition triad was found to be the mother’s level of education. It contributed about 17.62 percent in rate and 28.93 per cent in composition to the reduction in the Binary undernutrition and Undernutrition triad respectively. Result also shows that due to a change in the composition from the illiterate level to the higher level may play important role. Previous studies confirm our finding that the educational level of the mother is significantly associated with child undernutrition [21, 22]. It may be due to the reason that educated mothers have sufficient knowledge about child care, and are empowered to make better health decisions for their children. In addition, education imparts them with better job opportunities and increased household income which, in turn, enable them to provide more nutritious food options to their children and finance their healthcare needs [23, 24]. It is therefore not surprising that the governments have taken initiatives, both at the central and state levels, to promote girl’s education with various government-sponsored schemes such as—‘Beti Bachao Beti Padhao’, Sarva Shiksha Abhiyan, Balika Samridhi Yojna, etc.

Similar to other studies, we found that as compared to first-born children, children with higher birth orders were more likely to be malnourished [25, 26]. The reasons for this observation could be decreased household allocation of food and resources, more likelihood of higher order births being unwanted, reduced tendency towards antenatal and postnatal care and child check-up with the higher birth order, etc. [27, 28]. Several policies and programs have been launched by the Indian government to promote small family norms, including initiatives such as ‘Hum Do’ under the National Family Planning Programme, restricting financial and other maternity benefits for the third child, increasing awareness about family planning methods, and improving their availability.

Household wealth, as indicated by the wealth index, significantly influences both binary undernutrition and the undernutrition triad, contributing 11% to the rate of change in binary undernutrition and 32% to compositional changes in the undernutrition triad. In 1990, the all-India Poverty Head Count Ratio (PHCR) was estimated to be 48 per cent. In 2011–12, the PHCR was 22 per cent. A faster reduction in poverty since the mid-2000s helped India halve the incidence of poverty. This was a result of both economic growth as well as increased social spending on interventions such as the Mahatma Gandhi Employment Guarantee Act and the National Rural Health Mission [29]. This may explain the compositional change in wealth quintiles towards the richest category.

It was observed that as the economic condition of a family improved, children became less likely to face undernutrition as indicated by the lower levels of undernutrition in the middle, richer, and richest population groups. This relationship between economic status and undernutrition has been observed in the previous literature as well [30, 31]. It is probably due to the fact that poverty affects the availability of food supplies, the use of health services, and the availability of improved water facilities and sanitation practices, which are all important determinants of child undernutrition.

In this paper, the chief propensity factor, responsible for reduction in the Undernutrition triad, was found to be the preceding birth interval. Previous studies have found that a birth interval of less than 24 months is more commonly associated with child undernutrition [32]. This may be because a short birth interval may give women insufficient time to recover from the nutritional burden of pregnancy which may compromise the care received by the infant [33]. The mother’s BMI level also emerged as the primary factor, resulting in a significant 40% reduction in the Undernutrition triad. Studies have also highlighted that children born to anaemic mothers are at higher risk of developing anaemia [34, 35].

One of the important factors, which caused a difference of around six per cent in the Binary undernutrition during the inter-survey period was a place of residence. Researchers have observed that children’s nutritional outcomes in rural areas are affected by the low level of parental income, less hygienic environment, large family size, and short birth intervals. By contrast, urban children are at higher odds of a balanced meal, improved housing schemes, better healthcare services, access to potable water, higher availability of employment, and higher pay thereof [36]. Therefore, children from rural areas are more prone to undernutrition. In India, Nutrition-specific programs, such as Integrated Child Development Scheme (ICDS) and Mid-Day Meal (MDM) programs are being implemented in various states to support the nutritional needs of the children. The extent and equity of coverage of ICDS between 2006 and 2016, showed that the child-specific services grew from 10.4 per cent to 24.2 per cent which may have an impact on the reduction of child undernutrition [37]. Maharashtra’s nutrition mission, launched in 2005, aims to reduce undernutrition in all its forms. Similar initiatives have been undertaken by Madhya Pradesh, Karnataka, Gujarat, Uttar Pradesh, and Jharkhand [38].

The declining undernutrition levels in India highlight the efforts of the government for women and child development through various policies and programs. The elimination of binary and triad undernutrition in children is crucial to achieving many SDGs, such as Zero Hunger, Good Health and Well-Being, Reduced Inequalities, and the overarching ideal of leaving no one behind. It is a critical step towards ensuring that children around the world have a future that is healthier, better nourished, and sustainable. Present reduction in the Binary undernutrition and Undernutrition triad is a significant achievement for the Indian government, but a larger collective effort is still required to attain the global targets.

While carrying out this research, we failed to overcome all the limitations. We could not assess the direct impact of various nutrition programs responsible for the declining undernutrition during the inter-survey period. In addition to this, the role of dietary factors was not considered in this paper. It would have been interesting to know how dietary practices played a role in changing levels of child undernutrition during the 16-year study period. Future studies are recommended to explore the role of dietary practices in the reduction of child undernutrition in India.

## Supporting information

S1 Appendix Explanation of decomposition method. (DOCX)

S1 Table Sample characteristics of children aged 6–59 months in India, NFHS-3 and NFHS-5. (DOCX)

## List of abbreviations

NFHS– 3: National Family Health Survey– 3
NFHS– 5: National Family Health Survey– 5
WHO: World Health Organization
UNICEF: United Nations Children’s Fund
MOHFW: Ministry of Health and Family Welfare
SC: Scheduled Caste
ST: Scheduled Tribe
OBC: Other Backward Classes
SD: Standard Deviation
PHCR: Poverty Head Count Ratio
ICDS: Integrated Child Development Scheme
MDM: Mid-Day Meal
DHS: Demographic and Health Surveys
