# Supplementary material for: Decomposition analysis of the decline in binary and triad undernutrition among preschool children in India
Source: PLoS One. 2023 Oct 20;18(10):e0292322. doi: 10.1371/journal.pone.0292322 (PMC10588879; doi:10.1371/journal.pone.0292322)
Supplement: S1 Appendix — (DOCX) [file pone.0292322.s001.docx]

**S1 Appendix : Explanation of Decomposition Method**

The multivariate decomposition analysis of the logit or log odd of binary undernutrition and undernutrition tried is define as

Y=F(Xß)

The dependent variable is determined by the linear combination of predictors and their corresponding regression coefficients.

here Y is the dependent variable vector of size N × 1, X is the matrix of independent variables with dimensions N × K, and β is the coefficient vector of size K × 1. F(⋅) denotes a differentiable function that maps the linear combination of X(Xβ) to Y. By breaking down the overall differences between groups A and B into distinct components that represent compositional disparities (endowments) and variations in the effects of characteristics (coefficients), we can gain valuable insights into the specific factors that contribute to disparities observed between these groups.

Logit (A)-Logit (B)=F(X_A_ß_A_) –F(X_B_ß_B_)= [F(X_A_ß_A_) -F(X_B_ß_A_) ]_E_ +[F(X_B_ß_A_) -F(X_B_ß_A_) ]_C_

The E component is the portion of the overall decline in undernutrition that may be attributed to changes in the demographics of study participants across surveys. We used the logit link function, therefore the analysis of the multivariate logit data does not contain an error term. The C component denotes the portion of the overall decline in undernutrition that may be attributed to variations in the coefficients of variables between surveys. In order to identify significant factors that led to the decline in undernutrition, a p-value of 0.05 and the corresponding coefficient (B) with a 95% confidence range were utilised.
