# Supplementary material for: Decomposition analysis of the decline in binary and triad undernutrition among preschool children in India
Source: PLoS One. 2023 Oct 20;18(10):e0292322. doi: 10.1371/journal.pone.0292322 (PMC10588879; doi:10.1371/journal.pone.0292322)
Supplement: S1 Table — (DOCX) [file pone.0292322.s002.docx]

| Supplementary Table S1: Sample characteristics of children aged 6-59 months in India, NFHS-3 and NFHS-5 | | | | |
| --- | --- | --- | --- | --- |
| **Household level factors** | NFHS-3 | | NFHS-5 | |
| **Background Variables** | **%** | **N** | **%** | **N** |
| **Region** |  |  |  |  |
| North | 13.52 | 6,428 | 13.43 | 31,924 |
| Central | 28.15 | 7,846 | 26.59 | 42,733 |
| East | 27.35 | 5,941 | 26.93 | 34,026 |
| Northeast | 3.59 | 5,191 | 3.80 | 26,736 |
| West | 12.02 | 3,669 | 12.67 | 15,489 |
| South | 15.36 | 4,833 | 16.58 | 21,529 |
| **Place of Residence** |  |  |  |  |
| Urban | 24.27 | 12,326 | 26.22 | 34,605 |
| Rural | 75.73 | 21,582 | 73.78 | 1,37,832 |
| **Wealth Index** |  |  |  |  |
| Poorest | 25.25 | 6,156 | 24.46 | 46,773 |
| Poorer | 22.39 | 6,133 | 21.91 | 40,617 |
| Middle | 19.98 | 6,965 | 19.79 | 33,649 |
| Richer | 18.35 | 7,544 | 18.52 | 28,934 |
| Richest | 14.03 | 7,110 | 15.32 | 22,464 |
| **Religion** |  |  |  |  |
| Hindu | 78.94 | 24,415 | 79.84 | 1,26,407 |
| Muslim | 16.38 | 5,523 | 15.74 | 24,061 |
| Others | 4.68 | 3,970 | 4.41 | 21,969 |
| **Caste** |  |  |  |  |
| SC/ST | 30.05 | 10,904 | 33.48 | 70,817 |
| OBC | 40.57 | 11,325 | 43.32 | 65,245 |
| Others | 29.38 | 11,679 | 23.20 | 36,375 |
| **Household Members size** | |  |  |  |
| <5 | 21.42 | 7,975 | 25.77 | 45,065 |
| 5+ | 78.58 | 25,933 | 74.23 | 1,27,372 |
| **Mother level factors** |  |  |  |  |
| **Mothers education** |  |  |  |  |
| No education | 49.45 | 13,783 | 21.37 | 37,772 |
| Primary | 14.34 | 4,940 | 12.49 | 22,598 |
| Secondary | 31.34 | 12,694 | 51.16 | 89,204 |
| Higher | 4.86 | 2,491 | 14.98 | 22,863 |
| **Respondent's Current Age** |  |  |  |  |
| 15-24 | 39.59 | 12,168 | 30.71 | 49,028 |
| 25-34 | 51.51 | 18,468 | 60.62 | 1,05,410 |
| 35-49 | 8.90 | 3,272 | 8.67 | 17,999 |
| **Body Mass Index** |  |  |  |  |
| Thin | 39.79 | 11,467 | 19.82 | 32,527 |
| Normal | 53.2 | 18,972 | 60.73 | 1,07,827 |
| Overweight | 7.01 | 3,303 | 19.45 | 31,204 |
| **Media Exposure** |  |  |  |  |
| No exposure | 46.96 | 12,662 | 49.45 | 89,490 |
| Partial | 47.57 | 18,491 | 49.18 | 80,819 |
| Complete | 5.47 | 2,755 | 1.37 | 2,128 |
| **Preceding Birth**  **Interval (months)** | |  |  |  |
| First birth | 29.59 | 10,803 | 39.04 | 65,693 |
| <24 | 19.06 | 6,195 | 16.53 | 27,868 |
| >24 | 51.35 | 16,910 | 44.43 | 78,876 |
| **Age of the child (in months)** |  |  |  |  |
| 6-23 | 31.34 | 10,559 | 29.93 | 51,114 |
| 24-35 | 22.26 | 7,679 | 22.70 | 39,117 |
| >36 | 46.4 | 15,670 | 47.37 | 82,206 |
| **Sex of Child** |  |  |  |  |
| Male | 52.87 | 17,809 | 51.68 | 88,807 |
| Female | 47.13 | 16,099 | 48.32 | 83,630 |
| **Birth Order** |  |  |  |  |
| First order | 29.44 | 10,734 | 38.72 | 65,250 |
| 2-4 | 54.60 | 18,648 | 56.12 | 96,862 |
| More than 5 | 15.97 | 4,526 | 5.16 | 10,325 |
|  |  |  |  |  |
| **Diarrhoea** |  |  |  |  |
| No | 91.04 | 30,809 | 92.71 | 1,60,690 |
| Yes | 8.96 | 3,099 | 7.29 | 11,747 |
| **Fever** |  |  |  |  |
| No | 84.49 | 28,663 | 86.22 | 1,50,361 |
| Yes | 15.51 | 5,245 | 13.78 | 22,076 |
| **Total** |  | 33,908 |  | 1,72,437 |
